# Is There a Place for Versius (CMR) Robotic Platform in Children?

**DOI:** 10.3390/children13020290

**Published:** 2026-02-19

**Authors:** Marcin Losin, Andrzej Golebiewski, Piotr Czauderna

**Affiliations:** Department of Surgery and Urology for Children and Adolescents, Medical University of Gdansk, Dębinki 7, 80-211 Gdańsk, Poland

**Keywords:** robotics, pediatric surgery, versius

## Abstract

**Highlights:**

**What are the main findings?**

- The Versius robotic system was safely and successfully applied in pediatric patients as young as six years of age and with body weight as low as 15 kg, with no conversions to laparoscopy or open surgery.
- Early clinical outcomes demonstrated acceptable operative times, short hospital stay, effective postoperative pain control, and a complication profile comparable to early experiences with established robotic platforms.

**What are the implications of the main findings?**

- The Versius system represents a feasible and promising alternative robotic platform for selected pediatric urological and general surgical procedures.
- With further technical refinements and growing surgical experience, Versius may expand access to minimally invasive robotic surgery in pediatric populations, particularly in smaller children where current systems have limitations.

**Abstract:**

**Introduction:** Since its introduction in 1994, robot-assisted surgery has advanced significantly and has become a widely accepted tool in minimally invasive surgery. Over the past two decades, robotic technology has also been increasingly adopted in pediatric surgery. Currently, only two robotic systems are officially approved for pediatric use: the da Vinci surgical system and the Senhance system, both of which have certain limitations. To address these challenges, new robotic platforms such as the Versius system are being developed. **Materials and Methods:** Following approval from the institutional bioethics committee, a total of 14 pediatric patients underwent robotic-assisted surgery using the Versius robotic system between 10 June and 21 October 2024. Procedures included pyeloplasty, vascular hitch, and cholecystectomy. **Results:** Procedures with the Versius system were performed including children as young as six years of age and with body weight as low as 15 kg. All procedures were completed successfully without conversion to conventional laparoscopy or open surgery. No intraoperative complications were recorded. The overall postoperative complication rate was 21.4% (3/14 cases), including one anastomotic leak, one case of postoperative hematuria, and one case of postoperative ascites. **Discussion:** The Versius system represents a promising robotic platform for pediatric surgery, offering a different approach to robotic surgery through modularity, mobility, and compatibility with 5 mm instruments. However, several challenges remain, including prolonged setup and docking times, cable management issues, arm conflicts, and limited access to advanced instrumentation. Nevertheless, with ongoing technological development, robotic surgery is likely to play an increasingly important role in pediatric surgical care.

## 1. Introduction

Robotic surgery was introduced into urological practice with the AESOP system in 1994, marking the beginning of a new era in minimally invasive surgery. Subsequent technological developments, including the ZEUS system and, most notably, the da Vinci surgical system, have progressively enhanced surgical precision and operational efficacy [1,2,3,4]. Robotic-assisted surgery (RAS) has rapidly gained acceptance among adult patient populations, particularly in urological and gynecological procedures requiring deep pelvic access [2,3,4]. However, the adoption of robotic surgery in pediatric populations has been more limited and delayed, primarily due to technical challenges related to instrument miniaturization and ongoing concerns regarding the safety and efficacy of robotic interventions in younger and smaller patients [1,5,6,7].

The first reported cases of RAS in children appeared in 2001 and included procedures such as Nissen fundoplication and, shortly thereafter, pediatric pyeloplasty performed using the da Vinci system [1,5,6,7]. Since these early reports, more than seventy distinct robotic surgical techniques have been described in the pediatric surgical literature [5,7]. At present, the da Vinci system remains the principal robotic platform approved for pediatric use, demonstrating feasibility across pediatric populations older than six months and with body weight as low as 10 kg, when supported by careful preoperative planning and appropriate case selection [5,6,7,8].

Emerging technologies, such as the Versius robotic system (CMR Surgical, Cambridge, UK), aim to address key challenges limiting the broader adoption of robotic surgery in children, including the lack of appropriately sized instruments, anatomical constraints, and restricted working space [9]. The Versius platform is based on a modular design, with each robotic arm mounted on an independent mobile bedside unit, combined with an open-console configuration that differs from traditional closed-console systems [10]. The system is compatible with standard 5 mm laparoscopic ports and wristed instruments, allowing for flexible port placement and improved adaptability to pediatric anatomy. The open-console design supports surgeon ergonomics, facilitates communication with the operating room team, and enhances intraoperative teaching and training. Owing to these characteristics, the Versius system has demonstrated feasibility in preclinical and early clinical studies and represents a promising development in pediatric robotic surgery [1,11,12,13,14,15]. Therefore, the aim of this study was to present initial clinical experience and to assess the technical feasibility and perioperative safety of the Versius robotic system in pediatric surgery, and the study was designed as a platform-oriented feasibility assessment rather than a procedure-specific outcome analysis.

## 2. Materials and Methods

The primary objective of this prospective study was to assess the feasibility and safety of the Versius robotic system in pediatric patients, defined as the ability to complete planned procedures without conversion to laparoscopy or open surgery and without intraoperative complications. Secondary objectives included the evaluation of operative and docking times, early postoperative recovery parameters, and the incidence of postoperative complications. This study was designed as a prospective study evaluating the feasibility and early clinical outcomes of robotic-assisted pediatric surgery using the Versius robotic system (CMR Surgical, Cambridge, UK). The study protocol was reviewed and approved by the institutional bioethics committee prior to patient enrollment. All procedures were performed in accordance with the principles of the Declaration of Helsinki.

Between 10 June 2024 and 21 October 2024, a total of 14 consecutive pediatric patients were enrolled and underwent robotic-assisted surgical procedures using the Versius system at our institution. Inclusion criteria comprised pediatric patients requiring elective minimally invasive surgical treatment for urological or general surgical indications. No formal lower age or body weight limits were imposed; however, patient selection was based on anatomical feasibility, anticipated workspace, and overall clinical condition.

The cohort included eight patients undergoing dismembered Anderson–Hynes pyeloplasty for ureteropelvic junction obstruction, three patients treated with vascular hitch procedures for ureteropelvic junction obstruction caused by crossing vessels, and three patients undergoing cholecystectomy for symptomatic cholelithiasis. The distribution of procedures was defined prior to the description of the operative setup to provide appropriate clinical context for the technical aspects of the procedures.

The median age of patients was 10.5 years (range: 6–17 years), and the median body weight was 41 kg (range: 15–67 kg).

All procedures were performed using the Versius robotic system according to a predefined institutional robotic workflow protocol. Port placement, arm configuration, and the use of three or four robotic arms depending on the procedure and patient anatomy were tailored individually to optimize ergonomics and minimize the risk of arm collision, particularly in smaller patients. Because positioning depended on patient body proportions, the setup followed reproducible principles rather than fixed geometric coordinates.

In all cases, a 12 mm umbilical optical trocar was inserted using an open Hasson technique, and pneumoperitoneum was established at 10–12 mmHg.

A consistent robotic setup was applied throughout the series, with only minor variations dictated by the underlying pathology and the side of surgery. Bladder catheterization and gastric decompression with a nasogastric tube were routinely performed in all cases.

All patients were positioned supine. For renal procedures, the operating table was flexed with elevation of the side of interest by approximately 45°, whereas cholecystectomy procedures were performed in the standard flat supine position. During the initial cases, trocar spacing and bedside unit positioning were adjusted iteratively according to patient body size and instrument range of motion. Particular attention was paid to maintaining adequate distance between robotic arms to minimize external collisions, which proved more critical in smaller patients than in adolescents.

For renal procedures, three or four robotic arms were used depending on the side of surgery and the specific operative technique. Trocar placement followed a reproducible pattern, consisting of a central 12 mm optical port and two 5 mm robotic working ports positioned along the left midclavicular line. An additional assistant or lateral lumbar trocar (5 mm) was placed infraumbilically or laterally, depending on the side of the pathology and intraoperative requirements.

Consistent use of three robotic arms, with occasional addition of a fourth arm, reflected the modular concept of the Versius system and facilitated reproducibility of the operative setup. Classic three-arm docking was performed on the operative side; when a fourth arm was required, it was docked posteriorly on the side of the operated kidney.

For robotic cholecystectomy, patients were positioned supine, and four robotic arms were routinely employed. Trocar placement included a 12 mm umbilical camera port, two 5 mm robotic ports placed in the right and left upper quadrants, and a 5 mm subxiphoid trocar, in accordance with standard robotic cholecystectomy techniques.

Operative time was defined as the interval from skin incision to skin closure. Docking time was defined as the time required to position and connect the robotic arms to the trocars following port placement. Perioperative data collected included operative and docking times, intraoperative complications, conversion rates, postoperative opioid requirement, length of hospital stay, and postoperative complications. Complications were classified according to the Clavien–Dindo classification system, with major complications defined as grade III or higher.

Postoperative management followed standardized institutional protocols. Analgesia was provided using a multimodal regimen, with postoperative opioid use recorded as the number of days of administration. Patients were discharged once adequate pain control, tolerance of oral intake, and overall clinical stability were achieved.

## 3. Results

Robotic-assisted surgery using the Versius system was successfully completed in all 14 pediatric patients included in the study. The system was safely applied in children as young as six years of age and with body weight as low as 15 kg. No procedures required conversion to conventional laparoscopy or open surgery, and no intraoperative complications were recorded.

The median operative time across all procedures was 135 min (range: 70–255 min). Procedure-specific median operative times were 85 min (range: 70–116 min) for cholecystectomy, 130 min (range: 110–160 min) for vascular hitch procedures, and 179 min (range: 97–255 min) for dismembered Anderson–Hynes pyeloplasty with ureteropelvic anastomosis over a JJ stent. The median docking time was 17 min (range: 11–45 min). A progressive reduction in docking time was observed over the course of the study, with later procedures consistently demonstrating docking times between 11 and 19 min, reflecting a learning curve associated with system setup and arm positioning. When analyzed by procedure type, operative times differed according to procedural complexity, with the shortest median operative time observed for cholecystectomy and the longest for pyeloplasty. Despite procedural heterogeneity, all procedures shared common technical requirements related to port placement, arm positioning, and docking strategy, supporting the rationale for grouping them together as an assessment of platform feasibility rather than procedure-specific outcomes.

Postoperative recovery was generally rapid. The median length of hospital stay was 2 days (range: 2–5 days), with nearly 80% of patients discharged within two days following surgery. The median duration of postoperative opioid use was 2 days (range: 1–3 days), indicating effective pain control consistent with minimally invasive surgical approaches.

The overall postoperative complication rate was 21.4% (3 out of 14 cases). One major complication (7.1%) was recorded in a patient who underwent pyeloplasty and developed JJ stent dysfunction with subsequent anastomotic leak, which required secondary intervention consisting of cystoscopy with stent repositioning and prolonged urinary drainage. Minor complications (Clavien–Dindo grades I–II) occurred in two patients (14.3%). One patient experienced transient postoperative hematuria, which resolved spontaneously without additional treatment. Another patient developed postoperative ascites following cholecystectomy; this was attributed to pre-existing severe congenital heart failure and hepatic cirrhosis rather than the surgical procedure itself. All patients were followed after discharge, and no additional early complications were identified.

Detailed patient demographics, operative parameters, postoperative outcomes, and complications are summarized in Table 1 and Table 2.

## 4. Discussion

### 4.1. Interpretation of Results

This study was not designed to compare the Versius system with other robotic platforms or with conventional laparoscopy. As an initial feasibility report, its primary aim was to document early clinical experience and perioperative outcomes rather than to perform comparative effectiveness analyses. Therefore, operative times, complication rates, and length of hospital stay should be interpreted as descriptive benchmarks within the context of early system implementation.

The present results demonstrate that the Versius robotic system can be safely applied in selected pediatric patients undergoing urological and general surgical procedures. All planned operations were completed without conversion, and no intraoperative complications were observed. Operative times, hospital stay, and postoperative analgesic requirements were acceptable and consistent with expectations for an early institutional experience with robotic surgery. These findings suggest feasibility of the Versius platform in pediatric minimally invasive surgery and provide initial clinical reference points for its use in children [16].

Although the overall postoperative complication rate was 21.4%, only one complication was classified as major, and in two of the three cases, adverse events were unrelated to the robotic platform itself. This complication profile most likely reflects the early phase of system adoption and the associated learning curve rather than inherent limitations of the technology. For clarity, adverse events were analyzed according to their origin and categorized as procedure-related, patient-related, or platform-related. The anastomotic leak following pyeloplasty represents a procedure-related complication inherent to reconstructive urinary tract surgery rather than to the robotic approach itself. Transient postoperative hematuria was also considered a minor procedure-related event associated with upper urinary tract instrumentation. In contrast, postoperative ascites was classified as a patient-related complication, as it occurred in a child with pre-existing severe cardiac failure and hepatic cirrhosis and was not attributable to the surgical technique. No intraoperative failures, device malfunctions, or technical errors directly related to the robotic system were observed; therefore, no platform-related adverse events were identified in this series.

### 4.2. Significance of the Versius Platform in Pediatric Surgery

Robotic-assisted surgery has been increasingly adopted in pediatric practice due to advantages such as enhanced visualization, improved dexterity, and superior precision compared to conventional laparoscopy [17]. However, most established robotic platforms were originally designed for adult patients, which may limit their applicability in smaller children because of instrument size and workspace constraints.

In this context, the Versius system represents an alternative robotic platform with potential relevance for pediatric surgery. Its compatibility with 5 mm instruments and flexible arm positioning may facilitate adaptation to pediatric anatomy and confined operative fields. Rather than focusing on a single procedure, the present study adopted a platform-oriented approach, incorporating different operations that share common technical requirements related to port placement, docking strategy, and instrument handling. This strategy was chosen to explore system versatility during the early phase of clinical implementation.

### 4.3. Technical Challenges and Learning Curve

Despite these encouraging findings, several technical challenges associated with the Versius system were identified. Docking time was initially longer than typically reported for established robotic platforms, reflecting the need to position and coordinate multiple independent bedside units. With increasing experience, docking times decreased substantially, indicating a clear learning curve related to system setup and arm positioning.

Additional challenges included the need for meticulous preoperative planning to avoid robotic arm collisions, particularly in smaller patients with limited working space. The absence of real-time arm position registration may contribute to occasional arm conflicts requiring assistant intervention and potentially prolonging operative time. Furthermore, the presence of multiple columns and connecting cables can create spatial constraints in the operating room, affecting team mobility and access to the patient.

The open-console configuration introduces additional workflow considerations. While it supports intraoperative teaching and team communication, it requires the use of three-dimensional glasses and relies entirely on hand-controlled system functions due to the absence of foot pedals. These factors may reduce intuitiveness and contribute to surgeon fatigue during longer procedures. Collectively, these technical aspects may limit widespread adoption of the Versius system in high-volume pediatric centers until further refinements are introduced.

### 4.4. Practical Considerations and Lessons Learned

During early clinical implementation, several practical aspects proved essential for safe workflow. Wider initial spacing between ports reduced external robotic arm collisions, particularly in patients below 25 kg. Clear verbal communication between the console surgeon and the bedside assistant was necessary because the absence of haptic feedback required greater visual confirmation of safe movements, and it proved particularly important during instrument collisions and temporary arm clutching.

Frequent minor arm conflicts were observed during the first procedures and decreased markedly after standardizing arm angles and limiting excessive instrument articulation. We also found that careful planning of camera orientation before suturing significantly reduced unnecessary clutching and shortened reconstruction time. These observations represent practical workflow adaptations rather than technical limitations of the platform.

### 4.5. Study Limitations

This study has several limitations. The small sample size reflects an initial single-center experience and limits the statistical strength of the analysis. The heterogeneity of procedures restricts generalizability and precludes procedure-specific outcome comparisons. Finally, perioperative outcomes may have been influenced by the learning curve inherent to early system implementation. Larger multicenter studies with longer follow-up are needed to better define the role of the Versius system in pediatric surgical practice.

## 5. Conclusions

In conclusion, the Versius robotic system was safely and effectively applied in a selected group of pediatric patients undergoing urological and general surgical procedures. All operations were completed without conversion, with acceptable operative times, short hospital stay, and a complication profile consistent with early robotic experience. These findings indicate that the Versius platform appears feasible in selected case for pediatric minimally invasive surgery. Further experience and larger studies are required to better define its role in pediatric surgical practice.

## Figures and Tables

**Table 1:** Technical procedure details.

Feature	Pyeloplasty (*n* = 8)	Vascular Hitch (*n* = 3)	Cholecystectomy (*n* = 2)
Indication	UPJ obstruction with hydronephrosis	UPJ obstruction due to crossing vessel	Symptomatic gallstones
Typical side	Left (5)/Right (3)	Left (1)/Right (2)	Not side-dependent (gallbladder)
Patient position	Supine, flank elevated 45°	Supine, flank elevated 45°	Supine without flank elevation
Trocar pattern	Umbilical 12 mm + 2 × 7 mm + assistant 5–7 mm	Similar, with emphasis on lumbar port for vessel management	Umbilical 12 mm + three 7-mm ports
Robotic arms used	3–4, side-dependent	3–4, side-dependent	4 arms routinely

**Table 2:** List of procedures performed with Versius system.

NO.	Age (years)	Body Weight (kg)	Diagnosis	Procedure	Docking Time (min)	Operative Time (min)	Days of Opioid Use	Length of Stay (days)	Complications
1	17	67	Cholelithiasis	Cholecystectomy	26	70	1	3	0
2	14	48	Left hydronephrosis	Pyeloplasty	18	219	2	2	0
3	8	26	Left hydronephrosis	Pyeloplasty	13	205	2	2	0
4	11	59	Cholelithiasis	Cholecystectomy	23	70	1	2	ascites, correlated with congenital heart failure
5	17	54	Cholelithiasis	Cholecystectomy	12	116	1	2	0
6	8	34	Right hydronephrosis	Pyeloplasty	16	245	3	2	0
7	10	15	Left hydronephrosis	Vascular hitch	15	160	2	2	0
8	14	59	Left hydronephrosis	Pyeloplasty	45	255	2	2	0
9	13	55	Right hydronephrosis	Pyeloplasty	26	210	2	5	0
10	7	24	Left hydronephrosis	Pyeloplasty	15	133	2	2	JJ dysfunction, anastomosis leak
11	6	18	Right hydronephrosis	Pyeloplasty	13	127	2	2	hematuria
12	17	62	Right hydronephrosis	Vascular hitch	19	110	2	2	0
13	8	27	Left hydronephrosis	Vascular hitch	11	130	1	1	0
14	7	21	Left hydronephrosis	Pyeloplasty	19	97	2	2	0

## Data Availability

The original contributions presented in this study are included in the article. Further inquiries can be directed to the corresponding author.
